# Skeletal Muscle Index and Baseline Biomarkers in Atezolizumab-Treated Extensive-Stage Small-Cell Lung Cancer: A Real-World Cohort Study

**DOI:** 10.3390/curroncol33100639

**Published:** 2026-10-21

**Authors:** Taliha Güçlü Kantar, Tolga Doğan, Burcu Yapar Taşköylü, Atike Gökçen Demiray, Arzu Yaren, Tarık Şengöz, Gamze Gököz Doğu

**Affiliations:** 1 Department of Medical Oncology, Denizli State Hospital, Denizli 20070, Türkiye; dr_tolgadogan94@yahoo.com; 2 Department of Medical Oncology, School of Medicine, Pamukkale University, Denizli 20070, Türkiye; drburcuyapar@gmail.com (B.Y.T.); gokcenakaslan@gmail.com (A.G.D.); arzu_yaren@yahoo.com (A.Y.); ggd2882@gmail.com (G.G.D.); 3 Department of Nuclear Medicine, School of Medicine, Pamukkale University, Denizli 20070, Türkiye; tsengoz@pau.edu.tr

**Keywords:** small-cell lung cancer, skeletal muscle index, atezolizumab, albumin, lactate dehydrogenase, prognosis

## Abstract

Body composition and routinely available blood biomarkers may help characterize prognosis in extensive-stage small-cell lung cancer (ES-SCLC). We retrospectively evaluated 81 patients who received first-line carboplatin, etoposide, and atezolizumab. Baseline L3 skeletal muscle index (SMI) was derived from [18F]FDG PET/CT and classified using the Martin criteria. Overall survival (OS) was assessed using Kaplan–Meier and Cox regression. Of 80 classifiable patients, 33 (41.3%) had low SMI. Median OS was 10.51 months (95% CI, 7.98–14.26) and was 8.48 versus 14.26 months in patients with low versus normal SMI (log-rank *p* = 0.050). In the primary multivariable model, low SMI was associated with higher mortality (HR, 2.03; 95% CI, 1.07–3.86; *p* = 0.030); lower albumin and higher lactate dehydrogenase were also associated with mortality. In exploratory analyses, the low-SMI association persisted after post hoc adjustment for bone metastasis (HR, 2.16; 95% CI, 1.05–4.42; *p* = 0.035), although diagnostics suggested time-varying effects. Continuous SMI and alternative thresholds were not associated with OS, and low SMI was not associated with progression-free survival. Martin-defined low SMI may complement routine biomarkers in prognostic assessment, but its cutoff- and time-dependent association requires cautious interpretation and external evaluation.

## Simple Summary

People with extensive-stage small-cell lung cancer can have markedly different outcomes despite receiving the same treatment. Loss of skeletal muscle may reflect reduced physical reserve and could provide additional prognostic information. We measured skeletal muscle on routine baseline imaging scans in 81 patients treated with carboplatin, etoposide, and atezolizumab and also evaluated standard blood tests. Low muscle mass, defined using the Martin criteria, was associated with shorter overall survival, as were lower albumin and higher lactate dehydrogenase levels. However, the muscle finding varied according to the definition used and over time, and it was not associated with progression-free survival. These results suggest that muscle measurements may complement routine clinical and laboratory assessment, but they should not be used alone to select treatment and require confirmation in larger studies.

## 1. Introduction

Atezolizumab combined with carboplatin and etoposide improved survival in extensive-stage small-cell lung cancer (ES-SCLC) and became a standard first-line option [1,2]. Outcomes nevertheless remain heterogeneous. Clinical measures that capture both tumor burden and the patient’s physiologic reserve may help describe this variation, particularly in real-world populations that are less selected than trial cohorts.

Body mass index (BMI) does not distinguish skeletal muscle from adipose tissue. By contrast, the cross-sectional muscle area at the third lumbar vertebra (L3) can be measured on routine CT and normalized to height squared to obtain the skeletal muscle index (SMI) [3]. Low CT-derived muscle mass has been associated with treatment toxicity and shorter survival across solid tumors [4,5]. Threshold choice matters, however, because published definitions were developed in different populations and classify substantially different proportions of patients as having low SMI.

The Martin criteria use sex-specific thresholds and, in men, account for BMI, reducing the risk that excess body weight obscures clinically relevant muscle depletion. They were derived in a large oncology cohort and demonstrated prognostic value independent of BMI [4].

Low muscle quantity alone is not equivalent to clinical sarcopenia. Current consensus definitions place reduced muscle strength at the center of the diagnosis and use muscle quantity or quality as confirmatory evidence [6].

Systemic inflammation and nutritional deterioration are clinically relevant in SCLC because they may reflect the combined effects of tumor burden, cachexia, and impaired physiologic reserve. Routinely available markers such as hemoglobin, albumin, LDH, CRP, NLR, CAR, PNI, and mGPS capture partially overlapping inflammatory, nutritional, and disease-burden domains. Previous SCLC studies have linked low muscle mass with systemic inflammation and poor survival [7,8], while body-composition and blood-based indices have shown prognostic associations in patients receiving PD-1/PD-L1-based treatment [9,10,11]. Because these measurements are inexpensive and obtained before therapy, their combined evaluation may provide a practical framework for real-world risk stratification, although they should not be regarded as stand-alone treatment-selection tools.

We assessed baseline L3 SMI and routinely available blood biomarkers in patients with ES-SCLC treated with carboplatin, etoposide, and atezolizumab. OS was the primary endpoint.

## 2. Materials and Methods

### 2.1. Study Design and Patients

**Figure 1:**
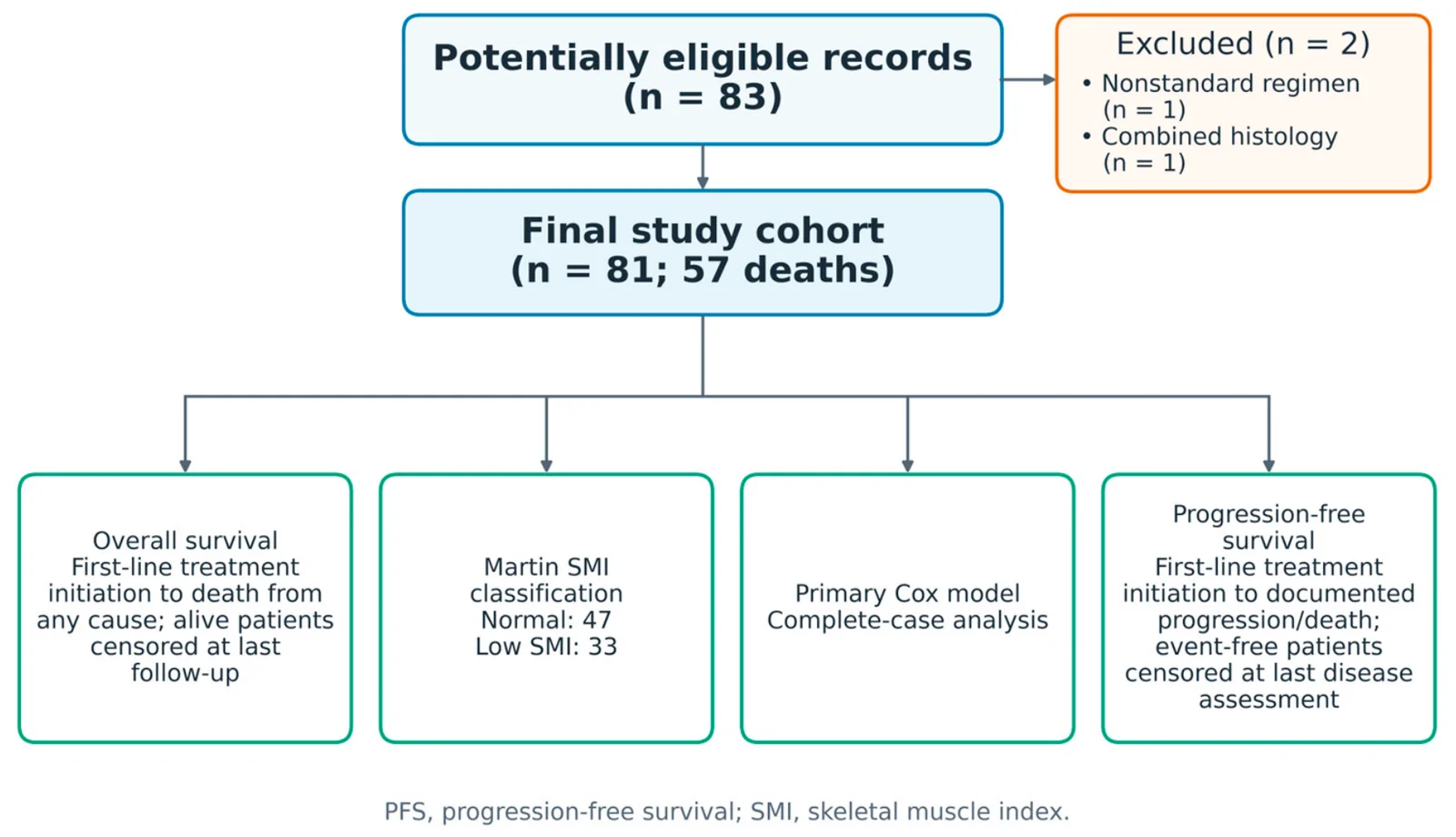
Study flow and analysis populations.

This single-center retrospective observational cohort included patients with ES-SCLC who started atezolizumab between May 2019 and January 2026. All included patients had an Eastern Cooperative Oncology Group performance status (ECOG PS) of 0–1 at treatment initiation. Ethical approval for this retrospective study was obtained from the Pamukkale University Non-Interventional Clinical Research Ethics Committee (meeting no. 12, 30 June 2026; document no. E-60116787-020-895520). The first-line regimen consisted of carboplatin, etoposide, and atezolizumab, followed by atezolizumab maintenance when clinically appropriate. Of 83 potentially eligible records, one patient treated with paclitaxel, carboplatin, and atezolizumab and one patient with combined small-cell and non-small-cell histology were excluded. The final cohort comprised 81 patients. The study flow and analysis populations are summarized in Figure 1.

### 2.2. Clinical Data

Recorded variables included age, sex, smoking exposure, height, weight, BMI, metastatic sites, number of atezolizumab cycles, radiotherapy, best documented response, subsequent systemic therapy, and vital status. Metastatic sites were non-mutually exclusive and were grouped as liver, adrenal gland, bone, lymph node, or other. Response categories were complete response, partial response, stable disease, progressive disease, and mixed response. Missing values were not imputed; available denominators are reported throughout.

### 2.3. Skeletal Muscle Assessment

Baseline skeletal muscle assessment was performed using the CT component of the [18F]FDG PET/CT examination. A single axial image at the third lumbar vertebral level was selected, and skeletal muscle was delineated using a predefined attenuation range of −29 to +150 Hounsfield units. The cross-sectional skeletal muscle area (cm^2^) was calculated and normalized to height squared to derive the skeletal muscle index (SMI, cm^2^/m^2^) [3]. PET/CT examinations were performed using a GE Discovery IQ 5-Ring PET/CT scanner (General Electric, Boston, MA, USA) equipped with a 16-slice CT component. The low-dose CT images were acquired with a slice thickness of 5 mm, and the same CT reconstruction algorithm was used for all patients. All measurements were reviewed by an experienced nuclear medicine physician. Grip strength and physical-performance measurements were not available in this cohort; we therefore use the terms low SMI and low skeletal muscle mass rather than sarcopenia. The Martin criteria were selected as the primary definition of low SMI, using the following externally defined thresholds: <43 cm^2^/m^2^ for men with BMI < 25 kg/m^2^, <53 cm^2^/m^2^ for men with BMI ≥ 25 kg/m^2^, and <41 cm^2^/m^2^ for women [4]. This externally defined framework was preferred to a cohort-derived cutoff in this modest retrospective sample and enabled clinically interpretable classification. One patient lacked information required for categorical classification. Because the Martin criteria were not developed specifically for SCLC, exploratory sensitivity analyses in the present study evaluated the Prado and previously reported SCLC-specific SMI thresholds, as well as continuous SMI, to assess the robustness of the primary findings.

### 2.4. Laboratory Biomarkers

Baseline laboratory measures used in the reported analyses were hemoglobin, absolute neutrophil count, absolute lymphocyte count, CRP, albumin, and LDH. Values recorded in inconsistent units were harmonized before analysis. NLR was calculated as neutrophils divided by lymphocytes, CAR as CRP (mg/L) divided by albumin (g/L), and PNI as albumin (g/L) + 5 × lymphocytes (10^9^/L). The mGPS was coded as 0 for CRP ≤ 10 mg/L, 1 for CRP > 10 mg/L with albumin ≥ 35 g/L, and 2 for CRP > 10 mg/L with albumin < 35 g/L.

### 2.5. Outcomes

Overall survival (OS) was calculated from the initiation of first-line treatment to death from any cause. Patients who remained alive were censored at the date of last follow-up.

Progression-free survival (PFS) was calculated from the initiation of first-line treatment to documented disease progression or death from any cause, whichever occurred first. Patients without progression or death were censored at the date of the last disease assessment. PFS was evaluated as an exploratory endpoint.

### 2.6. Statistical Analysis

Continuous variables are presented as medians and interquartile ranges (IQRs), and categorical variables as counts and percentages. The Mann–Whitney U test and Fisher’s exact test were used for between-group comparisons. Survival distributions were estimated with the Kaplan–Meier method and compared by the log-rank test. Median follow-up was estimated using the reverse Kaplan–Meier method. Candidate clinical, body-composition, and laboratory variables were first evaluated in univariable Cox proportional-hazards models. The primary model included age, Martin-defined low SMI, albumin, and LDH. Age was scaled per 10-year increase, albumin per 5 g/L increase, and LDH was log2-transformed so that its hazard ratio represented a doubling. An expanded sensitivity model additionally included liver metastasis and NLR. Because bone metastasis remained significant after false-discovery-rate correction in univariable analysis, a post hoc sensitivity model added bone metastasis to the primary covariates. An additional post hoc sensitivity model included BMI as a continuous covariate alongside the primary model covariates. Models with and without BMI were compared in the same patient sample. Models used complete cases. The proportional-hazards assumption was assessed using covariate-specific correlation tests between scaled Schoenfeld residuals and log event time. Where nonproportionality was detected, an exploratory extended Cox model included age × log(time) and low SMI × log(time) interactions, with time expressed in months. Benjamini–Hochberg false-discovery-rate adjustment was applied to a single family of 20 exploratory univariable Cox tests for OS. BH-adjusted *p* values are reported as q values; q < 0.05 was considered significant after correction. Tests were two-sided, and *p* < 0.05 was considered statistically significant. Analyses were conducted with Python(version 3.12)-based statistical libraries.

## 3. Results

### 3.1. Baseline Patient Characteristics

The cohort included 81 patients. All patients had an ECOG PS of 0–1 at treatment initiation. Sex was recorded for 80, of whom 75 (93.8%) were male. Median age was 64.0 years (IQR, 58.8–68.5), median smoking exposure was 50 pack-years (IQR, 40–56), and median BMI was 24.6 kg/m^2^ (IQR, 23.0–27.4); 34/81 patients (42.0%) were overweight or obese. Median L3 SMI was 47.4 cm^2^/m^2^ (IQR, 41.4–55.3). Among 72 patients with recorded metastatic-site data, lymph-node and bone metastases were present in 67/72 (93.1%) and 37/72 (51.4%), respectively; liver and adrenal metastases were present in 20/72 (27.8%) and 14/72 (19.4%), respectively. Overall demographic, anthropometric, disease, and treatment characteristics are summarized in Table 1. Eighty patients were classifiable by the Martin criteria: 33 (41.3%) had low SMI and 47 had normal SMI. The low-SMI group was older and had lower hemoglobin and a higher recorded frequency of adrenal metastasis. These comparisons are descriptive because multiple baseline characteristics were tested (Table 2).

### 3.2. Treatment and Response

Cycle count was available for 71 patients. The median total number of atezolizumab cycles was 9 (IQR, 5–13; range, 1–32), and 59/71 (83.1%) received at least four cycles. Radiotherapy information was available for 45 patients, and a response category was available for 53. Among response-evaluable patients, 35/53 (66.0%) had a complete or partial response and 37/53 (69.8%) achieved disease control. Table 3 reports the available denominators and comparisons by SMI group.

### 3.3. Overall Survival

**Figure 2:**
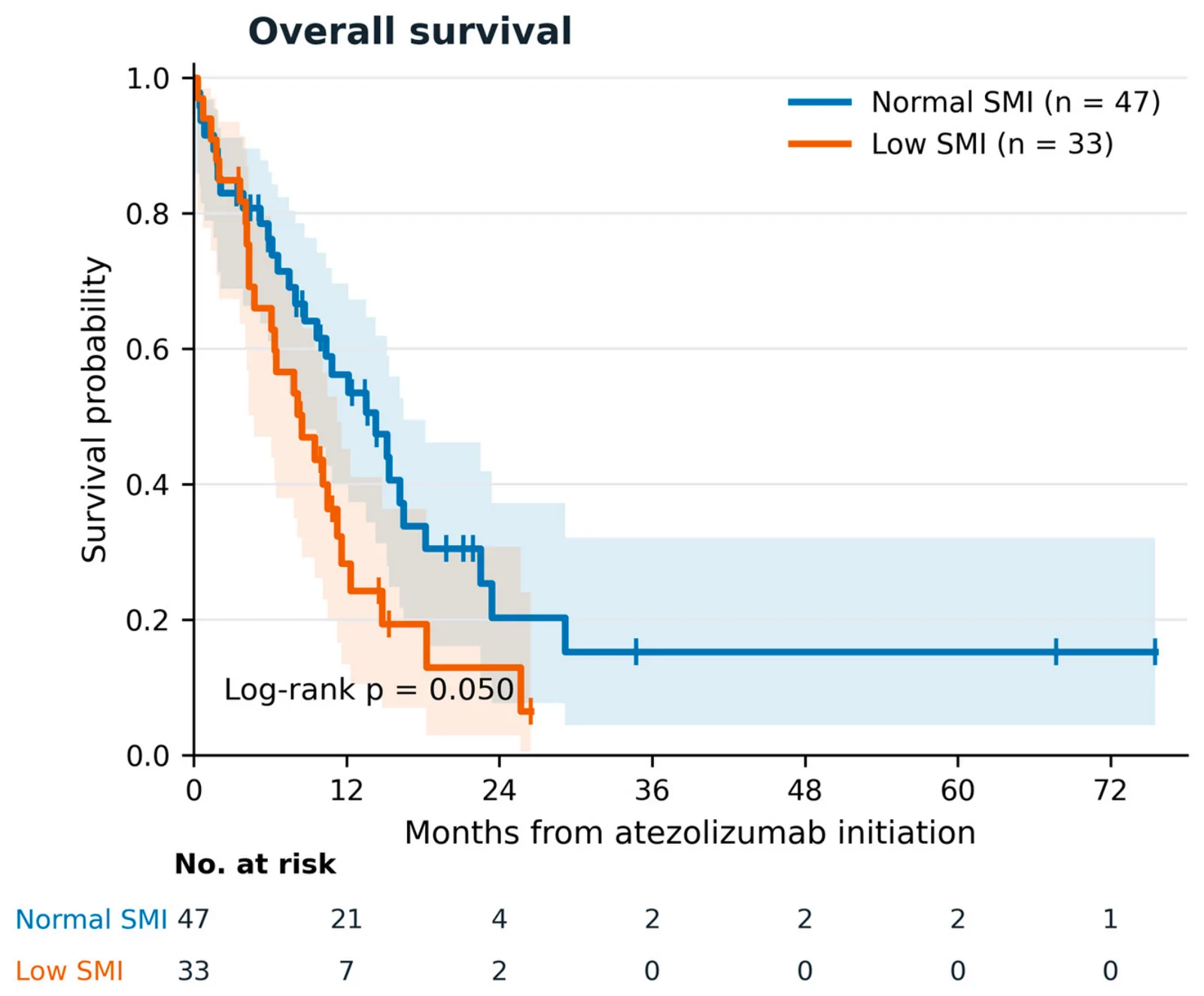
Kaplan–Meier estimates of overall survival according to Martin-defined SMI status.

Fifty-seven deaths occurred. Median follow-up was 21.16 months, estimated using the reverse Kaplan–Meier method. Median OS was 10.51 months (95% CI, 7.98–14.26) in the full cohort, 14.26 months in the normal-SMI group, and 8.48 months in the low-SMI group (log-rank *p* = 0.050; Figure 2). Martin-defined low SMI had a borderline association with mortality in univariable analysis (HR, 1.70; 95% CI, 0.99–2.90; *p* = 0.053; q = 0.132). Bone metastasis, lower albumin, higher LDH, higher CRP, higher NLR, higher CAR, and higher mGPS (per 1-point increase) were associated with OS before false-discovery-rate correction; only bone metastasis and LDH remained significant after correction (q = 0.023 for each).

**Figure 3:**
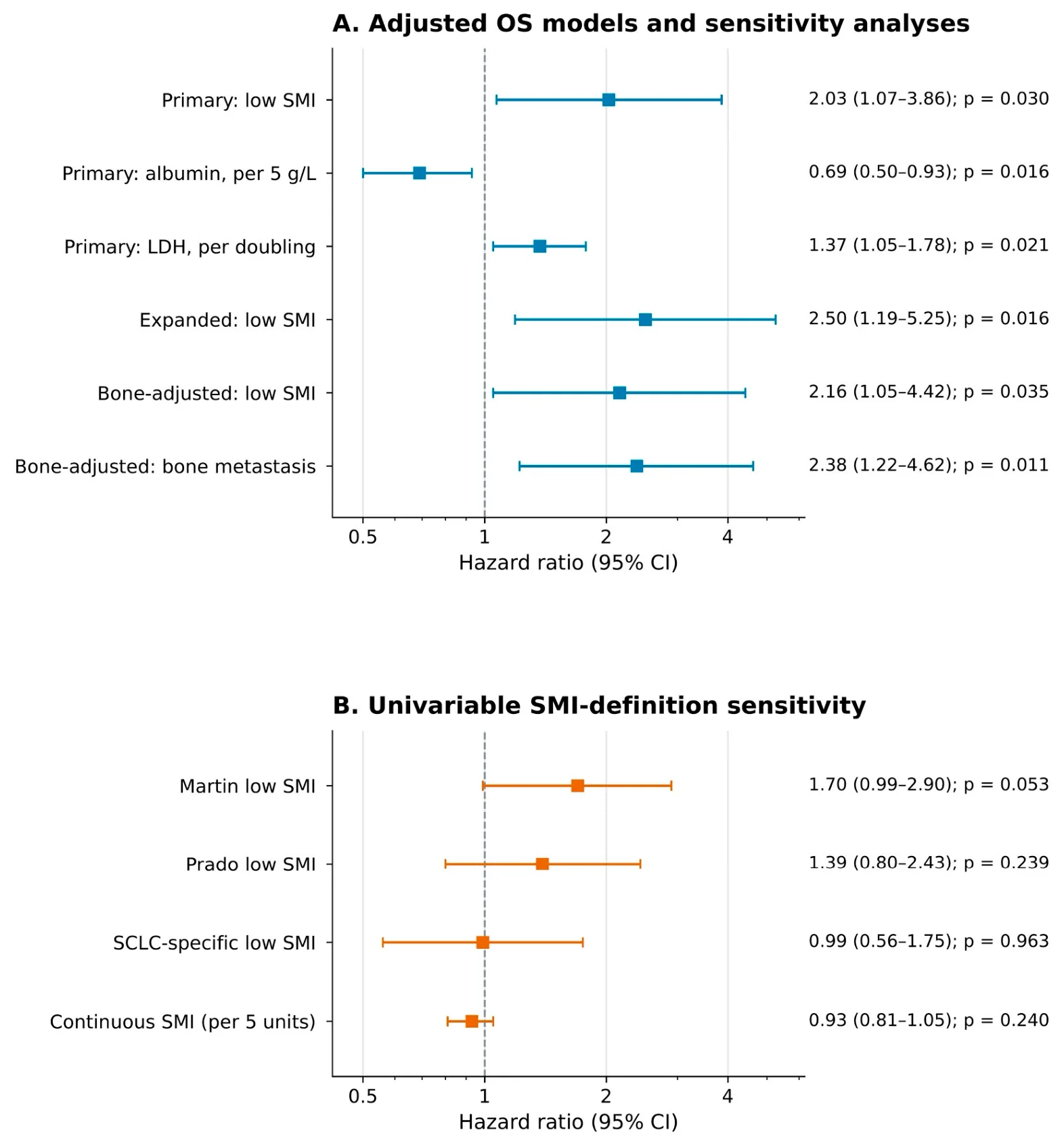
Cox-regression estimates for overall survival. (**A**) Selected adjusted hazard ratios from the primary model (n = 79; 55 deaths), the expanded model including liver metastasis and NLR (n = 71; 51 deaths), and the post hoc model adding bone metastasis to the primary covariates (n = 71; 51 deaths). (**B**) Univariable sensitivity analyses using alternative categorical SMI definitions and continuous SMI. For categorical definitions, hazard ratios compare low with normal SMI; the continuous estimate is per 5 cm^2^/m^2^ increase. CI, confidence interval; LDH, lactate dehydrogenase; NLR, neutrophil-to-lymphocyte ratio; OS, overall survival; SCLC, small-cell lung cancer; SMI, skeletal muscle index.

The primary multivariable model included 79 patients and 55 deaths. Low SMI was associated with higher mortality (HR, 2.03; 95% CI, 1.07–3.86; *p* = 0.030). Albumin was inversely associated with mortality (per 5 g/L: HR, 0.69; 95% CI, 0.50–0.93; *p* = 0.016), and LDH was positively associated with mortality (per doubling: HR, 1.37; 95% CI, 1.05–1.78; *p* = 0.021). The expanded model included 71 patients and 51 deaths. The association with low SMI persisted (HR, 2.50; 95% CI, 1.19–5.25; *p* = 0.016), whereas NLR, albumin, and LDH were no longer statistically significant. In the post hoc bone-adjusted model (n = 71; 51 deaths), low SMI remained associated with mortality (HR, 2.16; 95% CI, 1.05–4.42; *p* = 0.035), as did bone metastasis (HR, 2.38; 95% CI, 1.22–4.62; *p* = 0.011); albumin remained inversely associated (HR, 0.69; 95% CI, 0.49–0.98; *p* = 0.039), whereas the association between LDH and mortality was attenuated (HR, 1.10; 95% CI, 0.79–1.54; *p* = 0.572). In an additional BMI-adjusted sensitivity model (n = 79; 55 deaths), low SMI remained associated with higher mortality (HR, 2.03; 95% CI, 1.07–3.86; *p* = 0.031). Selected estimates are displayed in Figure 3A, whereas complete results for the primary and bone-adjusted models are presented in Table 4.

Covariate-specific tests based on scaled Schoenfeld residuals suggested nonproportionality for age (*p* = 0.008) and low SMI (*p* = 0.020), but not albumin (*p* = 0.874) or LDH (*p* = 0.231), in the primary model. In an exploratory extended model, the age × log(time) and low SMI × log(time) terms had *p* values of 0.011 and 0.014, respectively. The estimated low-SMI HR was 1.65 (95% CI, 0.83–3.28) at 3 months, 2.71 (95% CI, 1.39–5.26) at 6 months, and 4.45 (95% CI, 1.90–10.41) at 12 months. These post hoc estimates were imprecise and indicate that the conventional low-SMI HR should be interpreted as an average association over follow-up.

### 3.4. Progression-Free Survival

Median PFS was 7.06 months (95% CI, 5.26–8.90), with medians of 7.52 months (95% CI, 5.26–9.10) in the normal-SMI group and 6.24 months (95% CI, 4.21–8.90) in the low-SMI group (log-rank *p* = 0.470). In the adjusted model, low SMI was not associated with PFS (HR, 1.16; 95% CI, 0.66–2.04; *p* = 0.602). LDH remained associated with shorter PFS (per doubling: HR, 1.58; 95% CI, 1.17–2.14; *p* = 0.003).

### 3.5. Sensitivity Analyses for SMI Definition

Classification and OS separation changed when different SMI thresholds were applied. The Martin criteria classified 33/80 patients (41.3%) as having low SMI and yielded the largest observed separation. The Prado and SCLC-specific thresholds classified 61.3% and 70.0% as having low SMI, respectively, but neither definition showed a statistically significant difference in OS between the low- and normal-SMI groups. Continuous SMI was also not associated with OS (per 5 cm^2^/m^2^ increase: HR, 0.93; 95% CI, 0.81–1.05; *p* = 0.240) (Table 5).

## 4. Discussion

In this cohort of patients with ES-SCLC treated with carboplatin, etoposide, and atezolizumab, Martin-defined low SMI was associated with shorter OS after adjustment for age, albumin, and LDH. The association persisted when bone metastasis was added in a post hoc sensitivity model. Albumin and LDH were also associated with OS in the primary model, although the LDH estimate attenuated after adjustment for bone metastasis. The SMI result was not consistent across all analyses: continuous SMI was not associated with OS, and two alternative categorical thresholds produced little or no survival separation. Low SMI was also unrelated to PFS. Moreover, residual diagnostics suggested that the low-SMI effect varied over follow-up. These findings point to a possible prognostic role for muscle quantity, but the signal was dependent on how low SMI was defined and when the association was evaluated.

The borderline univariable association may partly reflect the aggressive natural history of ES-SCLC, which may limit the ability of baseline body-composition measures to discriminate outcomes during the early treatment period. The observed time-varying effect raises the possibility that the prognostic influence of low SMI becomes more apparent among patients surviving beyond this period. However, the small number of patients remaining at risk later in follow-up makes the time-specific estimates imprecise, and this interpretation should be considered exploratory.

The discordance between OS and PFS may also be clinically informative. Low SMI was associated with OS but not PFS, which may suggest that baseline muscle status reflects broader physiologic reserve rather than tumor-control duration alone. Lower muscle reserve could plausibly influence tolerance of ongoing therapy, vulnerability to intercurrent illness, or outcomes after disease progression; however, treatment toxicity, functional status, and post-progression survival were not specifically evaluated in this retrospective cohort. These explanations therefore remain hypothesis-generating rather than mechanistic conclusions.

The direction of the OS finding agrees with earlier SCLC reports. Kim et al. found shorter survival in patients with CT-defined low muscle mass [7], and a subsequent study connected muscle depletion with a stronger systemic inflammatory response [8]. A lung-cancer meta-analysis reached a similar overall conclusion, although the included studies varied in disease stage, treatment, imaging method, and cutoff [12]. Studies of PD-1/PD-L1 inhibitors have also reported worse outcomes in patients with low muscle mass, including a meta-analysis in non-small-cell lung cancer [9,13]. Our cohort adds information from patients receiving contemporary first-line chemoimmunotherapy, but it does not resolve the threshold problem. Differences in sample size, patient characteristics, and SMI thresholds may have contributed to the lack of significant differences in inflammatory markers between the SMI groups in our cohort. Baseline measurements alone also preclude assessment of changes in muscle mass and inflammation over time. These potential explanations could not be tested in the present study, and the absence of statistically significant differences should not be interpreted as evidence that no biological relationship exists.

That threshold problem is central to the interpretation. The Martin definition incorporates sex and BMI and was derived from a large, heterogeneous cancer population [4]. In the present cohort it identified fewer patients as having low SMI than either of the alternative definitions and produced the clearest separation in OS. A recent analysis across oncology cohorts likewise showed that clinical associations can change when different cutoffs are used [14]. Here, the absence of an association for continuous SMI makes it difficult to argue for a simple dose–response relationship. The cohort was also predominantly male, with only five women among patients with recorded sex, so the performance of female-specific thresholds cannot be assessed reliably. This also limits the generalizability of the findings to female patients.

Albumin and LDH contributed information that was not captured by SMI alone. Albumin may reflect inflammation, nutritional state, and hepatic function, whereas LDH is a nonspecific marker of tissue turnover and tumor burden. Both have been examined in recent ES-SCLC chemoimmunotherapy cohorts [10,15,16,17]. Their estimates became less precise when liver metastasis and NLR were added to the model. In the bone-adjusted sensitivity analysis, bone metastasis retained an adverse association and the LDH estimate attenuated, whereas low SMI remained associated with mortality. This pattern supports the relevance of disease burden while showing that the low-SMI estimate was not explained entirely by recorded bone metastasis. Shared biological information, fewer complete cases, and the modest number of events may all have contributed. The models also remain vulnerable to residual confounding. Although all patients had an ECOG PS of 0–1, residual differences in functional status, recent weight loss, comorbidity, and the full extent of disease may still have influenced both muscle mass and survival and were not fully captured in the adjusted models.

Using the CT component of baseline [18F]FDG PET/CT for skeletal muscle assessment exempts patients from the need for an additional imaging examination, as these images are already obtained for staging. L3 muscle measurements on routine CT are widely used in body-composition research [3], and PET/CT-derived muscle metrics have been explored in other malignancies [18]. However, the low-dose CT component of PET/CT may differ from diagnostic CT in slice thickness, reconstruction, contrast use, and patient positioning. Reader-dependent segmentation is another source of variation. Detailed acquisition and segmentation reporting, together with reproducibility testing, will be important before values from different centers can be compared.

Several other limitations affect the study. It was retrospective and conducted in a single center. The sample was modest, and missingness was substantial for radiotherapy, response, and metastatic-site variables. Response estimates were therefore based on an evaluable subset rather than the full cohort. SMI was assessed once, and muscle attenuation, longitudinal muscle loss, strength, and physical performance were not measured. Although all skeletal muscle measurements were reviewed by an experienced nuclear medicine physician, formal interobserver and intraobserver reproducibility assessments were not performed. Multiple exploratory comparisons increased the chance of false-positive findings despite false-discovery-rate adjustment. The bone-adjusted and time-varying analyses were post hoc. Proportional-hazards diagnostics indicated time-varying effects for age and low SMI, so the conventional Cox estimates summarize average associations rather than constant effects; the small number of events also limits flexible modeling of these changes.

The results do not support using SMI to select or withhold chemoimmunotherapy. They do suggest that a low muscle measurement on staging imaging can identify a group that may warrant closer nutritional and functional assessment. Interventions for cancer cachexia remain multimodal and individualized [19,20]. Whether improving muscle quantity or function changes survival in ES-SCLC will require prospective study.

## 5. Conclusions

Martin-defined low SMI, lower albumin, and higher LDH were associated with shorter OS in patients with ES-SCLC treated with atezolizumab-based chemoimmunotherapy. Low SMI remained associated with mortality in a post hoc model that additionally adjusted for bone metastasis, but diagnostics suggested that its effect varied over follow-up. The SMI association was not reproduced with continuous SMI or alternative thresholds, and low SMI was not associated with PFS. Thus, baseline muscle assessment may provide complementary prognostic information alongside routinely available clinical biomarkers, but the cutoff- and time-sensitive findings do not support SMI as a stand-alone treatment-selection tool. Larger multicenter studies using standardized imaging methods are needed to confirm its clinical utility.

## Figures and Tables

**Table 1:** Overall demographic and clinical characteristics of the study cohort.

Variable	Overall (n = 81)
Patients, n	81
Age, years	64.0 (58.8–68.5)
Male sex	75/80 (93.8)
Smoking exposure, pack-years	50 (40–56)
Height, cm	170 (165–172)
Weight, kg	70.0 (64.0–82.0)
BMI, kg/m^2^	24.6 (23.0–27.4)
Overweight or obesity (BMI ≥ 25 kg/m^2^)	34/81 (42.0)
L3 SMI, cm^2^/m^2^	47.4 (41.4–55.3)
Low SMI according to Martin criteria	33/80 (41.3)
Liver metastasis	20/72 (27.8)
Adrenal metastasis	14/72 (19.4)
Bone metastasis	37/72 (51.4)
Lymph-node metastasis	67/72 (93.1)
Other metastatic site	28/72 (38.9)
Total number of atezolizumab cycles, median (IQR); range	9 (5–13); 1–32
At least 4 atezolizumab cycles	59/71 (83.1)
Any radiotherapy	31/45 (68.9)
Objective response	35/53 (66.0)
Disease control	37/53 (69.8)

Continuous data are presented as median (IQR); the atezolizumab cycle count additionally includes the observed range. Categorical data are presented as n or n/N (%). Denominators reflect available data. BMI, body mass index; SMI, skeletal muscle index. One patient could not be classified by the Martin criteria because sex information was missing. Objective response was defined as complete or partial response; disease control included complete response, partial response, or stable disease.

**Table 2:** Selected baseline characteristics according to Martin-defined SMI group.

Characteristic	Overall	Normal SMI	Low SMI	*p*
Age, years	64.0 (58.8–68.5)	62.0 (57.5–66.0)	65.0 (60.0–71.0)	0.032
Male sex	75/80 (93.8)	44/47 (93.6)	31/33 (93.9)	1.000
Smoking exposure, pack-years	50 (40–56)	45 (40–50)	50 (40–58)	0.415
BMI, kg/m^2^	24.6 (23.0–27.4)	24.2 (23.6–29.2)	25.6 (22.0–26.5)	0.807
Overweight or obesity	34/81 (42.0)	16/47 (34.0)	18/33 (54.5)	0.107
L3 SMI, cm^2^/m^2^	47.4 (41.4–55.3)	54.9 (47.3–60.9)	40.1 (36.4–45.9)	<0.001
Liver metastasis	20/72 (27.8)	11/44 (25.0)	9/27 (33.3)	0.588
Adrenal metastasis	14/72 (19.4)	3/44 (6.8)	11/27 (40.7)	0.001
Bone metastasis	37/72 (51.4)	20/44 (45.5)	16/27 (59.3)	0.330
Lymph-node metastasis	67/72 (93.1)	40/44 (90.9)	26/27 (96.3)	0.643
Hemoglobin, g/dL	13.6 (12.2–14.9)	14.1 (12.4–15.1)	13.4 (11.8–14.2)	0.012
Albumin, g/L	39.4 (36.6–42.0)	39.2 (36.4–41.0)	39.8 (37.0–42.4)	0.822
LDH, U/L	268 (202–429)	254 (194–366)	298 (232–445)	0.184
NLR	3.94 (2.53–6.39)	4.15 (2.90–6.69)	3.34 (2.26–5.86)	0.320
PNI	46.8 (44.3–52.7)	46.8 (43.8–52.5)	47.0 (45.0–52.8)	0.590
mGPS ≥ 1	57/80 (71.2)	35/47 (74.5)	21/32 (65.6)	0.454

Data are median (IQR) or n/N (%). Denominators reflect available data. *p* values are descriptive and were not adjusted for multiplicity. The SMI comparison is expected because SMI defines the groups. BMI, body mass index; LDH, lactate dehydrogenase; mGPS, modified Glasgow Prognostic Score; NLR, neutrophil-to-lymphocyte ratio; PNI, prognostic nutritional index; SMI, skeletal muscle index.

**Table 3:** Treatment exposure, response, and survival status.

Characteristic	Overall	Normal SMI	Low SMI	*p*
Atezolizumab cycles	9 (5–13)	9 (5–13)	8 (5–12)	0.536
At least 4 atezolizumab cycles	59/71 (83.1)	36/43 (83.7)	23/28 (82.1)	1.000
Any radiotherapy	31/45 (68.9)	20/28 (71.4)	11/17 (64.7)	0.744
Thoracic radiotherapy	20/45 (44.4)	14/28 (50.0)	6/17 (35.3)	0.372
Brain radiotherapy	9/45 (20.0)	4/28 (14.3)	5/17 (29.4)	0.265
Objective response	35/53 (66.0)	23/32 (71.9)	12/21 (57.1)	0.375
Disease control	37/53 (69.8)	24/32 (75.0)	13/21 (61.9)	0.368
Post-atezolizumab systemic therapy	20/79 (25.3)	15/45 (33.3)	5/33 (15.2)	0.114
Death	57/81 (70.4)	30/47 (63.8)	26/33 (78.8)	0.216

Data are median (IQR) or n/N (%). Denominators reflect available data. SMI, skeletal muscle index.

**Table 4:** Multivariable Cox regression models.

Outcome	Variable	HR	95% CI	*p* Value
OS	Age, per 10 years	0.93	0.64–1.34	0.688
OS	Low SMI, Martin	2.03	1.07–3.86	0.030
OS	Albumin, per 5 g/L	0.69	0.50–0.93	0.016
OS	LDH, per doubling	1.37	1.05–1.78	0.021
OS, bone adjusted	Age, per 10 years	0.81	0.53–1.24	0.332
OS, bone adjusted	Low SMI, Martin	2.16	1.05–4.42	0.035
OS, bone adjusted	Albumin, per 5 g/L	0.69	0.49–0.98	0.039
OS, bone adjusted	LDH, per doubling	1.10	0.79–1.54	0.572
OS, bone adjusted	Bone metastasis	2.38	1.22–4.62	0.011
PFS	Age, per 10 years	0.97	0.68–1.37	0.847
PFS	Low SMI, Martin	1.16	0.66–2.04	0.602
PFS	Albumin, per 5 g/L	0.97	0.73–1.30	0.853
PFS	LDH, per doubling	1.58	1.17–2.14	0.003

CI, confidence interval; HR, hazard ratio; LDH, lactate dehydrogenase; OS, overall survival; PFS, progression-free survival; SMI, skeletal muscle index.

**Table 5:** Sensitivity of overall-survival results to the SMI definition.

SMI Definition	N	Low SMI, n (%)	Median OS, Normal (mo)	Median OS, Low (mo)	Log-Rank *p*	Univariable HR (95% CI)
Martin: men < 43 if BMI < 25, <53 if BMI ≥ 25; women < 41	80	33 (41.3)	14.26	8.48	0.050	1.70 (0.99–2.90)
Prado: men < 52.4; women < 38.5	80	49 (61.3)	14.26	10.12	0.237	1.39 (0.80–2.43)
SCLC literature: men < 55; women < 39 [7]	80	56 (70.0)	13.50	10.51	0.963	0.99 (0.56–1.75)

BMI, body mass index; CI, confidence interval; HR, hazard ratio; OS, overall survival; SCLC, small-cell lung cancer; SMI, skeletal muscle index.

## Data Availability

The data presented in this study are available from the corresponding author upon reasonable request. The data are not publicly available because they contain information that could compromise patient privacy.

## Abbreviations

The following abbreviations are used in this manuscript:

BMI	body mass index
CAR	C-reactive protein-to-albumin ratio
CRP	C-reactive protein
ES-SCLC	extensive-stage small-cell lung cancer
LDH	lactate dehydrogenase
mGPS	modified Glasgow Prognostic Score
NLR	neutrophil-to-lymphocyte ratio
OS	overall survival
PFS	progression-free survival
PNI	prognostic nutritional index
SMI	skeletal muscle index
